# Fat infiltration in the infarcted heart as a paradigm for ventricular arrhythmias

**DOI:** 10.1038/s44161-022-00133-6

**Published:** 2022-10-06

**Authors:** Eric Sung, Adityo Prakosa, Shijie Zhou, Ronald D. Berger, Jonathan Chrispin, Saman Nazarian, Natalia A. Trayanova

**Affiliations:** 1 Department of Biomedical Engineering, Johns Hopkins University, Baltimore, MD USA; 2 Alliance for Cardiovascular Diagnostic and Treatment Innovation, Johns Hopkins University, Baltimore, MD USA; 3 Department of Medicine, Division of Cardiology, Johns Hopkins Hospital, Baltimore, MD USA; 4 Division of Cardiology, Perelman School of Medicine, University of Pennsylvania, Philadelphia, PA USA

**Keywords:** Computational models, Ventricular tachycardia, Cardiology

## Abstract

Infiltrating adipose tissue (inFAT) has been recently found to co-localize with scar in infarcted hearts and may contribute to ventricular arrhythmias (VAs), a life-threatening heart rhythm disorder. However, the contribution of inFAT to VA has not been well-established. We investigated the role of inFAT versus scar in VA through a combined prospective clinical and mechanistic computational study. Using personalized computational heart models and comparing the results from simulations of VA dynamics with measured electrophysiological abnormalities during the clinical procedure, we demonstrate that inFAT, rather than scar, is a primary driver of arrhythmogenic propensity and is frequently present in critical regions of the VA circuit. We determined that, within the VA circuitry, inFAT, as opposed to scar, is primarily responsible for conduction slowing in critical sites, mechanistically promoting VA. Our findings implicate inFAT as a dominant player in infarct-related VA, challenging existing paradigms and opening the door for unexplored anti-arrhythmic strategies.

## Abstract (web summary)

In a combined computational and multi-center clinical study to investigate the effects of adipose fat tissue infiltration on ventricular arrhythmias, Sung et al. show that infiltrating adipose tissue, as opposed to scar, is the main cause of infarcted-related ventricular arrhythmias by slowing cardiac conduction in critical sites.

## Main

Sudden cardiac death is a major leading cause of mortality in the world. Life-threatening VAs greatly increase the risk of sudden cardiac death, especially in patients with prior myocardial infarction^1^. Despite advancements in anti-arrhythmic therapeutics and catheter ablation procedures^1,2^, VA prevalence and recurrence rates remain unacceptably high^3,4^, in part due to an incomplete understanding of the underlying substrate^5^. Thorough characterization of this substrate^6^ would improve existing, and inform novel, treatment strategies to decrease VA burden.

For decades, traditional dogma has maintained that disease-induced heterogeneous scarring and fibrosis infiltration in ventricles with ischemic (infarction) or non-ischemic cardiomyopathies forms the arrhythmia substrate^7,8^. Scar and fibrosis promote electrical wave conduction slowing and also uni-directional block^7^, creating a milieu conducive to arrhythmogenesis. Many clinical studies have used the visualization of ventricular scar on late gadolinium-enhanced cardiac magnetic resonance imaging (LGE-MRI) in localizing VA ablation targets^9–11^. However, these extensive efforts have failed to markedly improve VA recurrence rates, suggesting that scar characterization alone may be insufficient for identifying and eliminating VA.

inFAT is often observed in heart histological studies, penetrating into the myocardium and co-localizing with fibrosis^12–14^. However, it has remained an underappreciated aspect of post-infarct remodeling, and its clinical importance is ill-defined. Recent work from our group and others suggests that there is an association between inFAT and arrhythmia^15,16^. Clinically, inFAT is identifiable on contrast-enhanced computed tomography (CE-CT)^17,18^. However, because inFAT is intermingled with fibrosis^13^, the specific role of inFAT in VA propensity is difficult to discern. To date, no study has assessed the arrhythmogenic propensity of post-infarct inFAT versus scar in patients with ischemic cardiomyopathy or provided insight into whether scar and inFAT could synergistically combine to promote VA occurrence.

Here we present a combined prospective clinical and personalized mechanistic computational study aimed at comprehensively characterizing the role of inFAT versus scar in post-infarct VAs. In this two-center study, CE-CTs and LGE-MRIs were acquired for the first time concurrently from enrolled post-infarct patients undergoing VA ablation procedure, so that inFAT and scar distributions could be simultaneously visualized. As imaging and intraprocedural electroanatomic mapping (EAM) alone cannot distinguish which type of remodeling—inFAT or scar—is responsible for the aberrant electrical behavior in the ventricles, a personalized computational approach was employed to discern the mechanistic roles of inFAT versus scar in arrhythmogenesis. For this purpose, novel hybrid CT-MRI 3D heart models were constructed from the imaging scans of each patient. Our results challenge preexisting paradigms about infarct-related VA and implicate inFAT as a dominant player in post-infarct arrhythmias, thus opening the door for new strategies to effectively mitigate a patient’s arrhythmic burden.

## Results

**Fig. 1:**
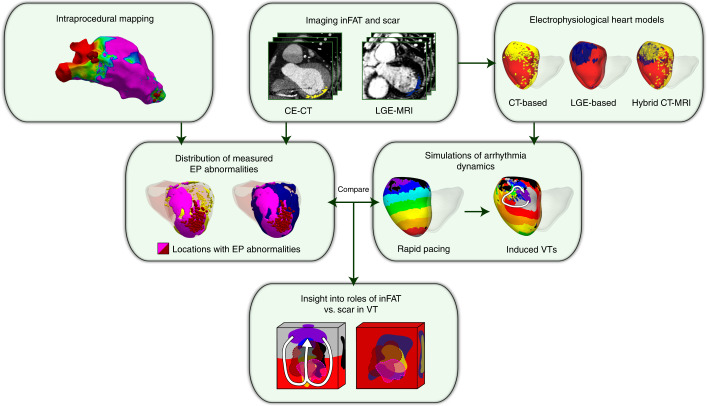
Overview of study. Schematic illustrating the combined two-center prospective clinical and mechanistic computational heart study. EP, electrophysiologcal.

Figure 1 provides an overview of the approach in our two-center prospective clinical and computational study. CE-CT and LGE-MRI images were acquired simultaneously for post-infarct patients who underwent ventricular tachycardia (VT) ablation (Fig. 1, first row, middle panel). Real-time electrical signals from substrate electroanatomical maps (EAMs) were acquired intraprocedurally during sinus rhythm (Fig. 1, first row, left panel). After image processing, three-dimensional (3D) distributions of scar on LGE-MRI and of inFAT on CE-CT were reconstructed within the geometry of the ventricles and co-registered with the EAM data to represent the distribution of measured electrophysiological abnormalities in the patient’s ventricles (Fig. 1, second row, left panel). These measured electrophysiological abnormalities were then compared with the results from simulations of VT dynamics (Fig. 1, second row, right panel) in three different electrophysiological heart models (LGE-based, CT-based and hybrid CT-MRI) created for each patient (Fig. 1, right side, top panel), so that the mechanistic role of inFAT versus scar in promoting VT could be dissected (Fig. 1, bottom panel).

### inFAT and scar distributions

**Fig. 2:**
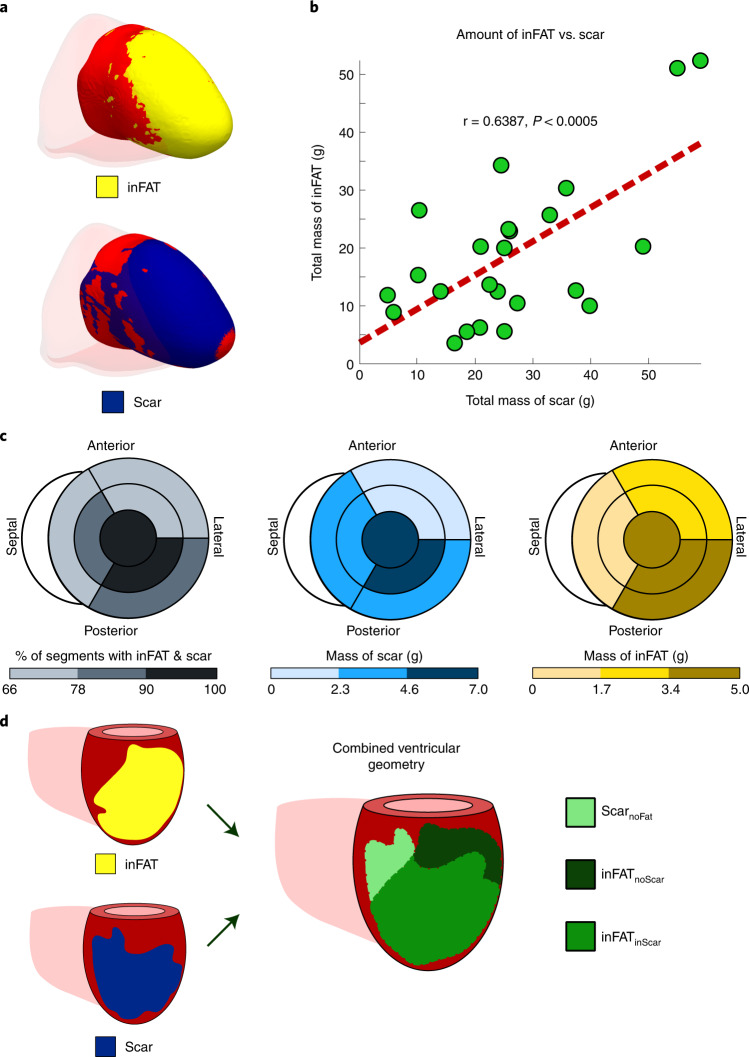
Distributions of inFAT and scar. **a**, Example of overlap between inFAT and scar distribution in a heart with an anterior infarct. **b**, Relationship between the total amount of scar and the total amount of inFAT across the patient cohort. Green dots represent data from individual patient hearts; in red is the line of best fit. **c**, Relationship between scar and inFAT distributions across different anatomical regions. The bullseye diagram shows a short-axis view (looking upward from below the heart). For each bullseye diagram, the outer and middle rings represent the three basal and middle segments, respectively (anterior/anterolateral, inferior/inferolateral and septal). The central segment represents the apex. The left panel shows the percentage of segments with both inFAT and scar; the middle and right panels show the mass of scar and inFAT across regions. **d**, Schematic of definitions for overlapping and non-overlapping regions between inFAT and scar. Three regions were defined: (1) inFAT and scar overlap (inFAT_inScar_); (2) scar not overlapping with inFAT (Scar_noFat_); and (3) inFAT not overlapping with scar (inFAT_noScar_).

Following the overview above, for each patient in the clinical study, first, 3D distributions of scar on LGE-MRI and of inFAT on CE-CT were reconstructed, using image processing, within the geometry of the patient’s ventricles. Figure 2 shows the relationship between inFAT and scar distributions across patient hearts. An example of overlap between inFAT and scar distributions in a heart with an anterior infarct is shown in Fig. 2a. The correlation between the total mass of inFAT and scar across reconstructed ventricular heart geometries was moderate (19.0 ± 13.0 g versus 26.3 ± 14.2 g, r = 0.639, *P* < 0.0005; Fig. 2b). inFAT and scar overlapped across hearts mostly in the apex (95.8%), the mid inferior/inferolateral (95.8%) and the basal inferior/inferolateral (87.5%) regions and the mid septum (83.3%). There was a lesser degree of overlap in the basal and mid anterior/anterolateral regions (75.0% and 70.8%) and in the basal septum (66.7%) (Fig. 2c, left).

Correlations between the inFAT and scar distributions were moderate in the apex (5.04 g and 6.80 g, r = 0.750, *P* < 0.0005) and in the mid anterior/anterolateral (2.36 g and 2.26 g, r = 0.621, *P* < 0.005), mid septal (1.52 g and 3.67 g, r = 0.578, *P* < 0.005), mid inferior/inferolateral (3.45 g and 6.99 g, r = 0.622, *P* < 0.005) and basal anterior/anterolateral (1.89 g and 0.64 g, r = 0.574, *P* < 0.005) regions. However, the amounts of inFAT and scar in the basal septum (1.07 g and 2.55 g, r = 0.304, *P* = 0.1489) and in the basal inferior/inferolateral regions (3.67 g and 3.37 g, r = 0.114, *P* = 0.5946) were not significantly correlated (Fig. 2c). Thus, these results indicate that there is a partial, but not complete, overlap between inFAT and scar distributions throughout the post-infarct left ventricle.

Given these results, we classified the post-infarct remodeling into three regions in a single combined ventricular geometry: (1) inFAT and scar overlap (inFAT_inScar_); (2) scar that does not overlap with inFAT (Scar_noFat_); and (3) inFAT that that does not overlap with scar (inFAT_noScar_). These three region definitions in the combined ventricular geometry are illustrated in Fig. 2d. These data also indicate that, to obtain mechanistic insights into the role of inFAT in VT, we would need to construct personalized heart models that are not only LGE-based or CT-based but also hybrid CT-MRI models that combine the two different types of remodeling.

### Distributions of electrophysiological abnormalities

Next, the distribution of measured electrophysiological abnormalities in the patient’s ventricles from the EAM data acquired during the ablation procedure was co-registered with the combined ventricular geometry of the patient containing the three regions: inFAT_inScar_, Scar_noFat_ and inFAT_noScar_. The goal here was to obtain distributions of measured electrophysiological abnormalities that could then be compared with the results of VT induction from mechanistic simulations to provide deeper insights.

**Fig. 3:**
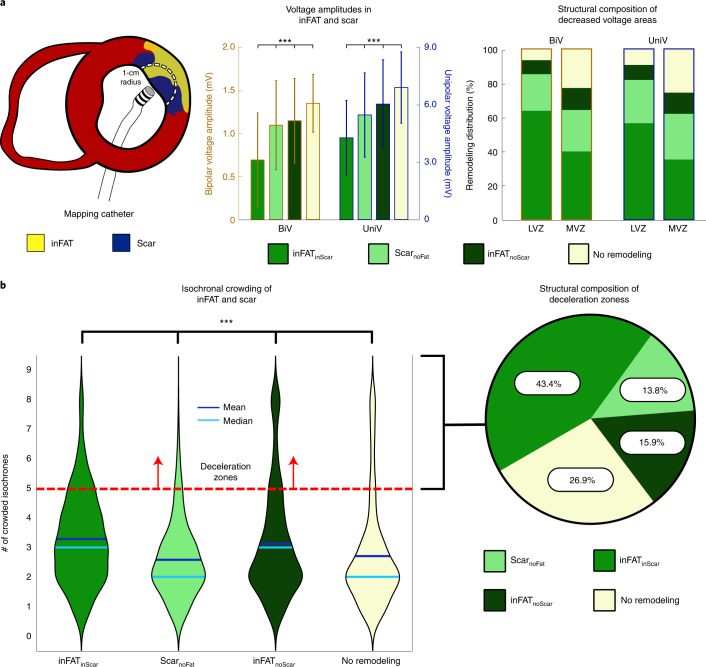
Distributions of clinically measured electrophysiological abnormalities in the ventricular geometries. **a**, Voltage amplitudes in the different types of regions. The left panel shows a schematic of how comparisons were made between EAM data and tissue content. An EAM catheter is used to record real-time, electrical signals along the myocardial surface. The circle of 1-cm radius represents the amount of myocardium around each EAM point that was considered. The middle panel shows the BiV and UniV in the regions of inFAT_inScar_ Scar_noFat_ and inFAT_noScar_. The right panel shows the scar and inFAT composition of decreased voltage areas (LVZs and MVZs) defined by both BiV and UniV cutoffs. **b**, Isochronal crowding within regions of inFAT and scar. The left panel shows violin plots that describe the distribution of isochronal crowding within regions of inFAT_inScar_, Scar_noFat_, inFAT_noScar_ and no remodeling. The right panel shows the structural composition of DZs defined as ≥5 crowded isochrones.

**Extended Data Fig. 1:**
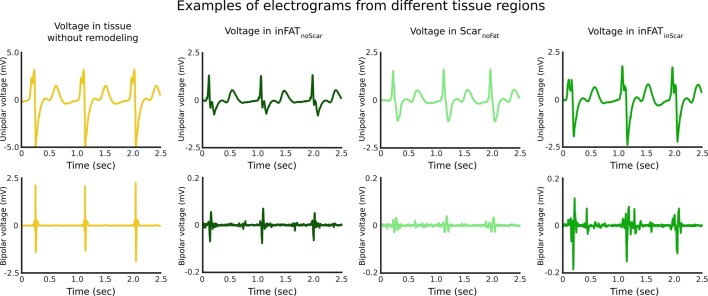
Examples of electrograms in different tissue regions. Top and bottom rows show unipolar and bipolar electrograms, respectively, recorded from tissue without remodeling, inFAT_noScar_, Scar_noFat_, and inFAT_inScar_. Note that the y-axis for both unipolar and bipolar electrogram recordings for tissue without remodeling is considerably different than the other y-axes. Electrogram recordings from inFAT_noScar_, Scar_noFat_, and inFAT_inScar_ generally showed diminished voltage amplitude as compared to tissues without remodeling. Note that the bipolar electrogram recorded in inFAT_inScar_ exhibits a complex pattern of activity, consistent with the presence of conduction abnormalities.

We first represented the distributions of bipolar and unipolar voltage amplitudes (BiV and UniV), which are the most commonly obtained electrical measurements from EAM^19^, in these three types of regions. Examples of electrograms across the different tissue regions are shown in Extended Data Fig. 1. Figure 3a presents the distribution of voltage amplitude for each type of region in the reconstructed ventricles across all patients; a schematic of how this was accomplished is shown in the left panel of Fig. 3a (see Methods for further details). Voltage amplitudes were significantly lower (*P* < 0.0005) in inFAT_inScar_ (BiV: 0.69 ± 0.55, UniV: 4.28 ± 1.94) than in Scar_noFat_ (BiV: 1.09 ± 0.52 mV, UniV: 5.46 ± 2.19 mV), inFAT_noScar_ (BiV: 1.15 ± 0.55, UniV: 6.02 ± 2.31 mV) and tissue without remodeling (BiV: 1.35 ± 0.33, UniV: 6.88 ± 1.86 mV) (Fig. 3a, middle). Low-voltage zones (LVZs) and medium-voltage zones (MVZs) were defined using BiV and UniV cutoffs from the literature^1^. LVZs existed predominantly in inFAT_inScar_ rather than Scar_noFat_, inFAT_noScar_ or tissue with no remodeling (BiV: 63.9% versus 21.4% versus 8.2% versus 6.5%; UniV: 56.6% versus 25.3% versus 8.7% versus 9.3%) (Fig. 3a, right). Similarly, MVZs typically localized to inFAT_inScar_ and less often to Scar_noFat_, inFAT_noScar_ and tissue without remodeling (BiV: 40.0% versus 24.3% versus 12.9% versus 22.8%; UniV: 35.3% versus 26.8% versus 12.4% versus 25.5%). Thus, inFAT_inScar_ encompasses most low-voltage areas and exhibits lower-voltage amplitudes than Scar_noFat_ and inFAT_noScar_.

**Extended Data Fig. 2:**
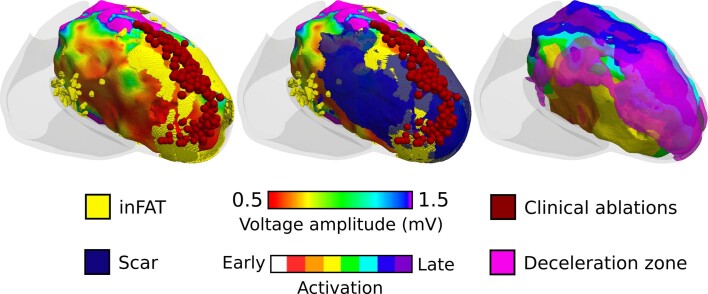
Example showing how deceleration zones localize to regions of inFAT. Left panel shows a voltage map, catheter ablations (dark red), and inFAT co-registered. Middle panel shows the left panel with the overlayed scar distribution. Right panel shows the sinus rhythm activation sequence with overlayed deceleration zones (pink). Deceleration zones localize to regions where inFAT is present.

We next determined the localization, in the ventricular geometries, of the following clinical measurements: (1) clinical ablation lesions, delivered at locations in the substrate deemed most likely to terminate VT, and (2) deceleration zones (DZs), which are regions of electrophysiological abnormalities with crowding of isochrones (equally spaced activation time windows) indicative of conduction slowing^20^. Per patient, the amount of inFAT in ablation lesions correlated with the amount of scar in the lesions (6.59 g and 5.25 g, r = 0.734, *P* < 0.05), suggesting that ablations, which primarily targeted sites with electrophysiological abnormalities conducive to VT, often ended up in inFAT_inScar_. Figure 3b shows that inFAT_inScar_ and inFAT_noScar_ regions both exhibited greater numbers of crowded isochrones (DZs) than did Scar_noFat_ and tissue without remodeling (3(2) and 3(2) versus 2(1) and 2(1), *P* < 0.0005) (Fig. 3b). DZs primarily encompassed inFAT_inScar_, inFAT_noScar_ or tissue without remodeling but less so Scar_noFat_ (43.4%, 15.9% and 26.9% versus 13.8%) (Fig. 3b). The presence of inFAT (both inFAT_inScar_ and inFAT_noScar_) was positively associated with the presence of DZs; there was a stronger relationship between inFAT_inScar_ and DZs (odds ratio (OR) = 1.69 (1.64, 1.74), *P* < 0.0005) than between inFAT_noScar_ and DZs (OR = 1.42 (1.36, 1.47), *P* < 0.0005, Fisher’s exact test). An example of DZs localizing primarily to inFAT_inScar_ is shown in Extended Data Fig. 2. On the contrary, the absence of inFAT was negatively associated with the presence of DZs (Scar_noFat_ OR = 0.45 (0.43, 0.46), *P* < 0.0005; tissue without remodeling OR = 0.83 (0.81, 0.86), *P* < 0.0005, Fisher’s exact test). These results indicate that regions with inFAT, whether overlapping with scar or not, were prone to slowed conduction. Thus, regions with inFAT exhibited the most substantial pro-arrhythmic abnormal electrophysiological properties.

The measured electrophysiological abnormalities described above were next compared with the results from personalized computational modeling so that the separate contributions of scar and inFAT to arrhythmogenesis could be mechanistically dissected.

### Arrhythmogenicity of inFAT versus scar

To address this, we created three different ventricular heart models per patient: one reconstructed from LGE-MRI, another from CE-CT and a hybrid CT-MRI model; each model incorporated electrophysiological properties as described in the Methods. Inducibility of VTs after rapid pacing was examined. Because rapid pacing was delivered in each model from a number of widely distributed sites in the ventricles, the manifested VTs provide a comprehensive assessment of the patient-specific substrate arrhythmogenic susceptibility arising from the different types of remodeling^21^.

**Fig. 4:**
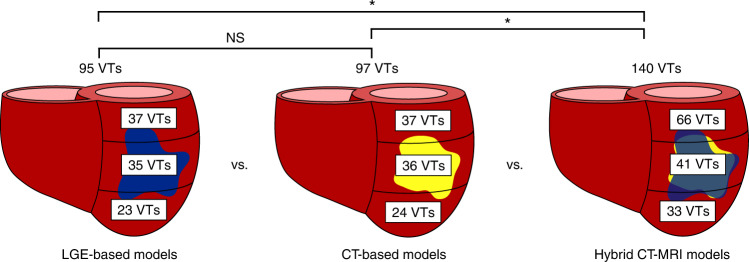
Arrhythmogenicity of inFAT versus scar. Number and distribution of VTs across the three different heart models. NS, not significant.

**Extended Data Fig. 3:**
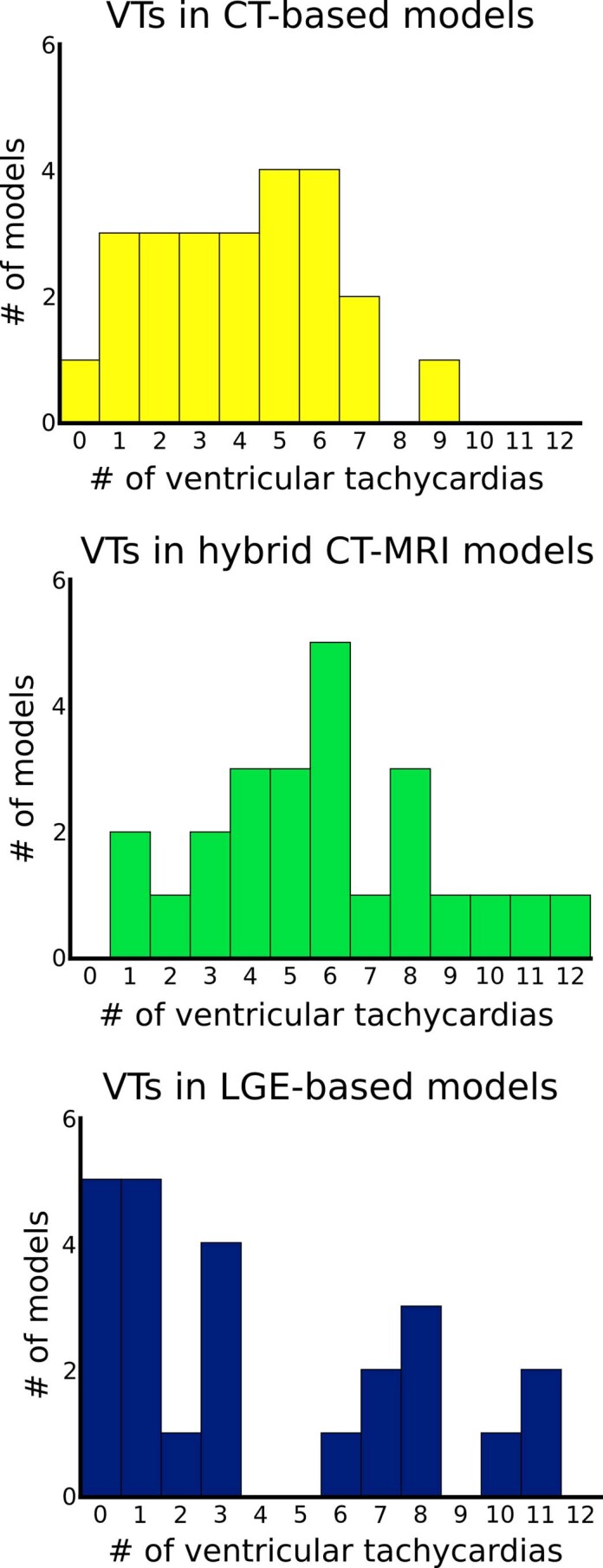
Distribution of ventricular tachycardias across the different heart models. The x-axis shows the number of VTs and the y-axis shows the number of models with that number of VTs. (n = 95 CT-based models, n = 97 LGE-based models, n = 140 hybrid CT-MRI models). CT: computed tomography, LGE: late gadolinium-enhanced, VT: ventricular tachycardia.

We first investigated the VTs manifesting within the inFAT-based substrate in heart models reconstructed from CE-CT images and those manifesting within the scar-based substrate in heart models based on LGE-MRIs. The inFAT-based substrate exhibited similar arrhythmogenic propensity as the scar-based substrate in terms of the total number of VTs induced (4.0 ± 2.2 versus 4.0 ± 3.8 VTs, *P* > 0.05). Figure 4 presents the number of VTs across the base, middle and apex of different heart models. Extended Data Fig. 3 provides additional detail on the distribution of VTs across heart models. The presence of VTs in these general regions was also similar between the two substrates (inFAT versus scar: 37 versus 37 basal VTs, *P* > 0.05; 36 versus 35 mid VTs, *P* > 0.05; 24 versus 23 apical VTs, *P* > 0.05) (Fig. 4). Thus, inFAT alone exhibits a propensity for VT that is similar to that of scar alone.

We next investigated the VTs manifesting in the hybrid CT-MRI heart models, which are the complete representations of the post-infarct substrate (both inFAT and scar). These hybrid models allow us to determine whether there are interactions between the inFAT and scar distributions that affect arrhythmogenicity in the combined substrate. Rapid pacing induced a total of 140 VTs in the combined inFAT-and-scar substrate (66 basal VTs, 41 mid VTs and 33 apical VTs) across all heart models (Fig. 4). There were significantly more VTs in the combined inFAT-and-scar substrate than in the scar-based substrate alone (5.8 ± 2.9 versus 4.0 ± 3.8 VTs, *P* < 0.05) and in the inFAT-based substrate alone (5.8 ± 2.9 versus 4.0 ± 2.2 VTs, *P* < 0.05).

We then assessed whether the amount of inFAT or scar in all hearts was associated with the number of VTs induced in the hybrid CT-MRI models. In a multivariable Poisson regression model, inFAT, but not scar, was significantly associated with the total number of VTs induced in these models. For every 5.55-g increase in the total amount of inFAT, there was a 10% increase in the total number of VTs induced (*P* < 0.05). The total amount of scar was not significantly associated with the number of induced VTs (*P* > 0.05). This indicates that, for a given heart, increasing amounts of inFAT, rather than scar, result in an increased arrhythmic burden in the combined substrate.

### Most critical VT isthmuses comprise both inFAT and scar

**Fig. 5:**
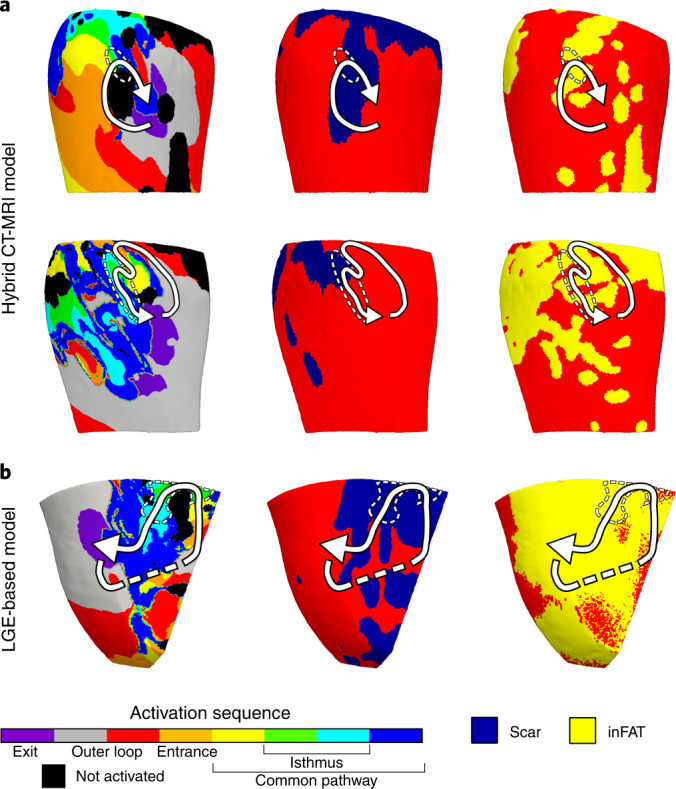
Most VT critical isthmuses comprise both inFAT and scar. **a**, Example of two VT circuits from hybrid CT-MRI models with isthmuses consisting of both inFAT and scar. Arrows trace the VT circuit from the exit site to the common pathway. Dashed circles denote the location of the critical isthmus. **b**, Example of a VT circuit from an LGE-based heart model. The inFAT distribution, superimposed from CE-CT, is displayed in the right panel.

We next determined what were the types of regions where VTs perpetuated in the personalized heart models. First, we evaluated the hybrid CT-MRI model VTs that manifested in the combined inFAT-and-scar substrate. In these hybrid CT-MRI heart models, most VT circuits (a VT circuit comprised the exit, outer loop, entrance, common pathway and isthmus^22^) encompassed both inFAT (2.89 ± 2.46 g) and scar (4.58 ± 3.17 g). Critical VT isthmuses, which represent the ideal ablation targets within the inner part of the circuit, more often consisted of inFAT_inScar_ (101/140 (72.1%) VTs) than Scar_noFat_ (22/140 (15.7%) VTs), inFAT_noScar_ (13/140 (9.3%) VTs) or tissue without remodeling (4/140 (2.9%) VTs) (*P* < 0.0005). Figure 5a presents two examples of VT circuits in hybrid CT-MRI models with isthmuses consisting of both inFAT and scar.

We also investigated the critical VT isthmuses in the LGE-based models. Although these models do not explicitly represent the inFAT distribution, we hypothesized that critical VT isthmuses in these models would localize to regions of the scar that overlapped with inFAT (when superimposed from CE-CT). We found that most critical isthmuses (66/95 (69.5%) VTs) contained scar and also overlapped with inFAT as superimposed from CE-CT (1.83 ± 1.47 g and 0.44 ± 0.89 g, r = 0.49, *P* < 0.0005). These results indicate that regions of the scar with inFAT are more likely to harbor critical sites of the VT circuit than just scar alone. Figure 5b shows an example of a VT circuit from an LGE-based heart model along with the scar and superimposed inFAT distributions. Altogether, these data indicate that most critical VT isthmuses contain inFAT, and, thus, inFAT may play a direct mechanistic role in promoting re-entrant VT.

### Comparing VTs in the heart models with clinical EAM data

To determine the mechanistic involvement of inFAT in re-entrant VT, we investigated whether inFAT, as opposed to scar, could be the factor primarily responsible for the pro-arrhythmic conduction abnormalities in regions with greater VT propensity. Accordingly, we evaluated the relationship between inFAT or scar located within the VT circuitry in the simulations and the EAM-defined electrophysiological abnormalities (locations of ablations and DZs).

First, we examined whether the amount of inFAT or scar in the region encompassing the VT circuit in the hybrid CT-MRI heart models was associated with the volume of clinically measured pro-arrhythmic conduction abnormalities. Larger amounts of inFAT in regions that encompassed the VT circuits were associated with larger volumes of ablated tissue (β = 0.175, *P* < 0.0005) and DZs (β = 0.336, *P* < 0.05). In contrast, the amount of scar in these regions was not associated with the volume of ablated tissues (β = −0.03, *P* = 0.16) or DZs (β = −0.221, *P* = 0.06). Hence, in regions with greater VT propensity, the amount of inFAT, rather than scar, is a primary determinant of the amount of electrophysiological and conduction abnormalities.

Next, we evaluated the association between LGE-based heart model VTs and EAM data. In these LGE-based models, larger amounts of inFAT (as superimposed from CE-CT) in the region encompassing the VT circuit were associated with a larger volumes of ablated tissue (β = 0.377, *P* < 0.0005) and DZs (β = 0.698, *P* < 0.005). The same relationship was not true for the amount of scar in the region encompassing the VT circuit and the volumes of ablated tissue (β = −0.067, *P* = 0.28) or DZs (β = 0.214, *P* = 0.10). Collectively, these results indicate that, in regions with higher VT susceptibility, inFAT, but not scar, is the primary source of pro-arrhythmic conduction abnormalities.

### inFAT promotes abnormal conduction in critical VT sites

Lastly, to better understand how inFAT promotes re-entry within the VT circuit, we investigated the VT component in which inFAT and EAM data (ablations and DZs) overlapped. Within VT circuits in hybrid CT-MRI heart models, the overlap between inFAT and DZs occurred more within the common pathway (33.7%) and entrance (27.5%) than in the outer loop (11.1%) and exit (12.6%). Similarly, clinically ablations were more likely to overlap with inFAT in the common pathway (20.3%) and entrance (19.3%) than in the outer loop (9.7%) and exit (11.1%) of the model VT circuits.

**Fig. 6:**
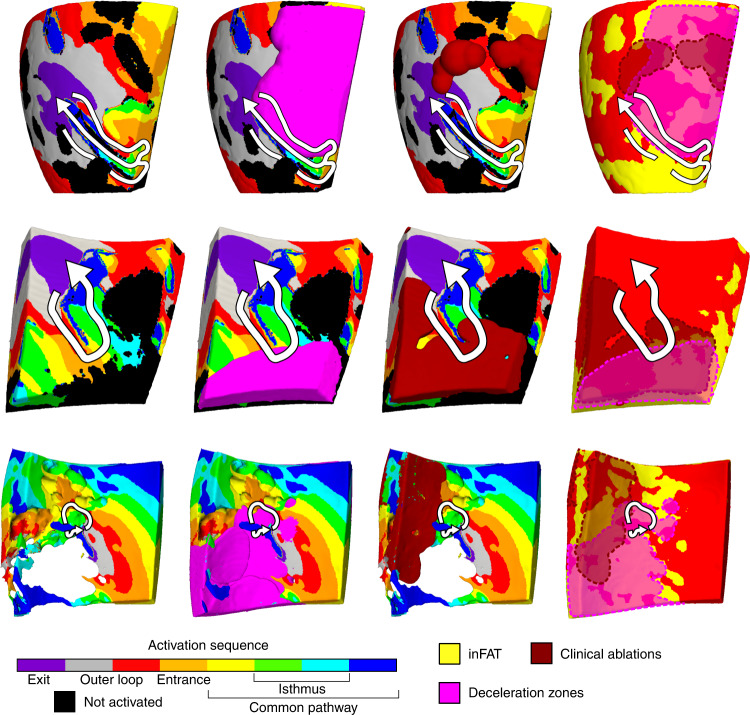
inFAT promotes conduction abnormalities in critical sites of the VT circuitry. Three examples of VT circuits in hybrid CT-MRI heart models with the overlapping clinical ablations and DZs are displayed. For these three VT circuits, there was inFAT but little to no fibrosis present. Arrows trace the re-entrant pathway from the exit site to the end of the common pathway. The pink regions denote the DZs, and the dark red denotes the estimated volume of the ablation lesion. Non-activated tissues were omitted for the bottom VT circuit to better visualize the intramural activation sequence.

Figure 6 provides three examples depicting where ablations and DZs from EAM localize within VT circuits in hybrid CT-MRI models. For these examples, there was little to no fibrosis present within the region of the VT circuit. The electrophysiological abnormalities primarily localized to the areas of inFAT within the entrance and common pathways of the model VT circuit (Fig. 6). Conduction slowing and non-uniform propagation at these specific locations of the circuit are essential for the maintenance of re-entrant VT^23–25^. Hence, these results highlight how inFAT mechanistically promotes re-entrant VT by slowing conduction within critical sites of the VT circuit.

## Discussion

In this first combined multi-center prospective clinical and personalized computational study, we elucidate the role of inFAT versus scar in infarct-related VT propensity. Through imaging and intraprocedural EAM data, we discovered that inFAT exhibits greater pro-arrhythmic electrophysiological abnormalities than scar. Using personalized heart models, we demonstrate that inFAT, rather than scar, is the primary driver of substrate arrhythmogenic propensity and is frequently present together with scar in the VT isthmus, which is the ideal target for ablation therapy. Lastly, using a combined analysis of both the clinical EAM and mechanistic simulation data, we identify that inFAT, and not scar, is the primary source of arrhythmogenic conduction slowing that is present at critical sites of the VT circuitry. Our results redefine conventional ‘known’ paradigms about infarct-related arrhythmias and implicate inFAT as a major player in VT, thus opening the door for potential new anti-arrhythmic strategies.

Our study challenges prevailing paradigms for infarct-related VT. The infarct scar distribution has long been considered the major source of pro-arrhythmic structural remodeling in the substrate for VT^7,8^. The canonical teaching is that heterogeneous scarring and electrical changes over time in the chronic infarct transform into the substrate necessary for VT. However, not all parts of the scar are necessarily arrhythmogenic^26^, and not all post-infarct patients will develop VT. inFAT is also present in the post-infarct substrate and develops ~3 years after infarction^18^, a timescale similar to how long it takes most VTs to manifest. Concordantly, inFAT seems to be present more often in patients with VT^16^. inFAT is not a passive bystander for several reasons. First, inFAT predicts arrhythmic burden independently of other important clinical factors. Specifically, inFAT has been shown to be an independent predictor of a composite outcome, including mortality and ventricular arrhythmias^27^ and VT recurrence^28^. In the current study, we demonstrated that larger amounts of inFAT predicted an elevated VT burden in hybrid CT-MRI models; in contrast, an increase in the amount of scar was not associated with increased VT burden. Second, inFAT is commonly present across sites critical for VT. In our study, we first uncovered how regions of inFAT, even in the absence of scar, were associated with greater conduction slowing. Furthermore, we demonstrated how most critical VT isthmuses in both hybrid CT-MRI and in LGE-based heart models were in locations with inFAT, meaning that regions with inFAT have an enhanced susceptibility for VT. This finding is consistent with previous studies that demonstrated that critical VT sites were often in the vicinity of inFAT^15,16,28^. Thus, inFAT is not a passive bystander but plays a major role in infarct-related VT.

Our study further establishes a mechanism by which inFAT promotes re-entrant VTs. Cardiac adipose tissue can exhibit pro-inflammatory and paracrine effects^29^ that result in conduction slowing and repolarization abnormalities^30,31^. Specifically in the ventricles, myocardial tissue in the vicinity of inFAT exhibits slowed conduction speed^32^ and electrogram abnormalities^16,28^. Consistently, we discovered that inFAT exhibits smaller voltage amplitudes and more conduction slowing than scar, indicating that inFAT is likely to be more pro-arrhythmic than scar. Furthermore, we identified that, within heart model VT circuits, larger amounts of inFAT, but not scar, were significantly associated with larger volumes of clinical EAM-defined conduction abnormalities. These conduction abnormalities overlapped with inFAT specifically at the VT entrance and common pathway, which are critical sites for ablation. It is well-established that conduction slowing at these specific regions of the circuit are essential for maintenance of re-entrant VT^23–25^. Hence, inFAT mechanistically promotes re-entrant VT by slowing conduction in the critical components of the circuit.

inFAT could promote conduction slowing through three primary mechanisms: (1) structural changes to myocardial architecture, (2) paracrine effects and (3) adipocyte–myocyte electrical coupling. Adipocytes are non-excitable, and their presence alone will directly alter the normal myocardial architecture. This altered myocardial structure disrupts normal electrical propagation and will slow conduction^7,8^. Paracrine effects of adipokines secreted by inFAT are also another possible mechanism of conduction slowing. However, no data currently exist on the secretome profile of inFAT. Epicardial adipose tissue (EAT), a tissue type that is distinct from inFAT, is well-known to be metabolically active and secretes a number of adipokines^29,33^. These adipokines have been established to alter myocyte electrophysiology and cause conduction slowing in atrial tissues^31,34,35^. Although EAT and inFAT are separate entities, there may be some similarities in the adipokines secreted by both tissue types. Studies are needed to investigate the relationship between EAT and inFAT in the post-infarcted ventricles. A third possibility that has been hypothesized is electrotonic coupling between adipocytes and myocytes, which could, in theory, alter myocyte excitability via changes to sodium channel inactivation and increase risk of spontaneous depolarizations^34^. However, there has been no proof of adipocyte–myocyte heterocellular coupling in intact hearts. Hence, we would hypothesize that inFAT causes conduction slowing through two primary mechanisms: formation of a structural barrier to conduction and release of paracrine factors that alter ventricular myocyte electrophysiology.

We envision several potential advancements to clinical management resulting from our findings. From a procedural perspective, non-invasive identification of inFAT on imaging could help shorten procedure times and improve ablation efficacy. Intraprocedural EAM of the post-infarct substrate is a labor-intensive process that does not always successfully elucidate the critical VT sites, because not all regions with electrical abnormalities identified on EAM are necessarily arrhythmogenic^26^. However, our study implies that regions with inFAT are likely to harbor critical VT sites. Thus, time and effort could be devoted to mapping these specific arrhythmogenic areas with inFAT instead of the entire infarct. In addition, knowledge of how the inFAT is distributed would also affect how ablations are delivered. Adipose tissue is a known inhibitor of ablation lesions formed by radiofrequency energy. If critical VT sites are found deep within the inFAT distribution, then more aggressive ablation strategies should be employed at these areas, involving either more lesions delivered or more advanced ablation techniques^36^. From a non-procedural perspective, we envision new strategies that prioritize decreasing the extent of inFAT in post-infarct VT patients. Reduction of cardiac adiposity is possible via medical therapies and may work synergistically with current standard-of-care treatments for VT. For instance, pharmacological agents such as sodium-glucose co-transporter 2 (SGLT2) inhibitors can reduce the amount of cardiac adiposity^37,38^ and have been shown to decrease arrhythmic events in randomized controlled trials^39,40^. Decreasing inFAT would eliminate substrate available to sustain VTs and, thus, reasonably result in decreased arrhythmia burden.

In conclusion, we present, to our knowledge, the first combined prospective clinical and personalized computational study that assesses the role of inFAT versus scar in infarct-related VT. We demonstrate that inFAT, as opposed to scar, is a primary determinant of arrhythmic burden and mechanistically promotes VT. These findings redefine conventional wisdom regarding infarct-related VT pathogenesis, implicating inFAT as a new, important player. We envision that this new knowledge will motivate novel, patient-specific therapeutic strategies that target inFAT to better address infarct-related VT burden.

## Methods

### Patient cohort

Patients were enrolled in a prospective, multi-center study at two institutions approved by our institutional review boards (IRBs) (IRB study 831270). Written informed consent was obtained from all patients. Inclusion criteria included history of myocardial infarction, history of VT and having undergone VT ablation. All patients enrolled in the study had cardiac CE-CT and LGE-MRI imaging acquired. Furthermore, all patients received a substrate-based ablation (patient characteristics and procedure details in Supplementary Methods). All images were assessed for quality; patients whose images were of insufficient quality were excluded from the study. Based on these criteria, a total of 24 patients were enrolled: 11 from Johns Hopkins Hospital and 13 from the University of Pennsylvania Hospital, from 2019 to 2021. All data were acquired under the guidance of our IRBs and appropriately de-identified.

EAM data from the CARTO mapping system were obtained for patients from the Johns Hopkins Hospital. However, one patient did not have CARTO mapping data and was not amenable for offline analysis.

### Image processing

**Extended Data Fig. 4:**
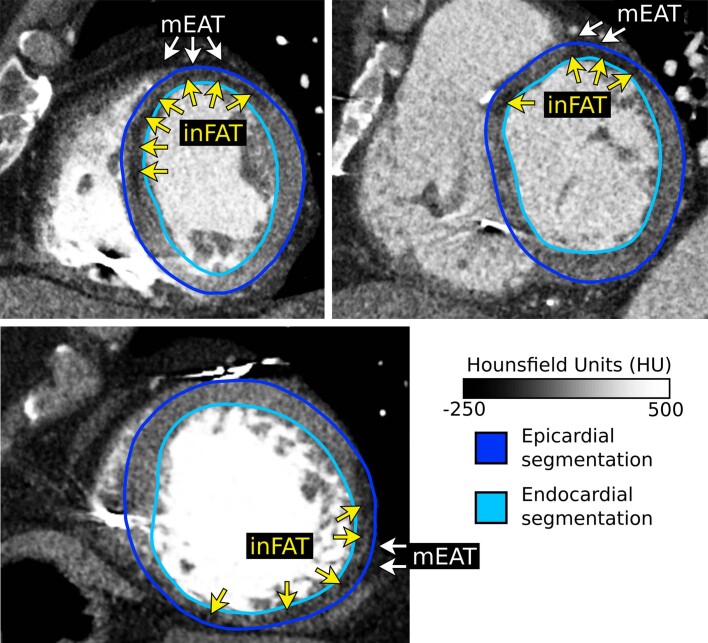
Representative examples showing the distinction between inFAT and mEAT. Epicardial and endocardial segmentation contours are shown. Epicardial contours were specifically drawn to exclude any overlying mEAT. Adipose tissue appears as hypoattenuation on CT (darker areas) and can be distinguished from blood pool and myocardium readily. Across all CE-CT images (n = 24), inFAT was always found within the segmented myocardium whereas mEAT is found overlying the epicardium. CE-CT: contrast-enhanced computed tomography, inFAT: infiltrating adipose tissue, mEAT: myocardial epicardial adipose tissue.

CE-CT images were acquired, using the same protocol as described previously^16^, at a resolution of 0.428–0.625 × 0.428–0.625 × 1.0–3.0 mm. Eighteen two-dimensional (2D) LGE-MRI images at a resolution of 1.3 × 1.3 × 5–8 mm and six 3D LGE-MRI images at a resolution of 1.3 × 1.3 × 2.5 mm were also obtained, using a 1.5-T MRI scanner and the same protocol as previously described^41^. All images were resampled into short axis at an isotropic resolution of 0.35 × 0.35 × 0.35 mm for ease of processing and segmentation. Myocardium in both CE-CT and LGE-MRI images were semi-automatically segmented as reported in previous publications^41–43^. In brief, landmark control points were placed along the left ventricular endocardium and epicardium. From these points, 3D endocardial and epicardial surfaces were automatically generated using the variational implicit method^44^. For all images, overlying EAT was carefully excluded from the segmentation. Extended Data Fig. 4 presents examples of CT scans showing how EAT was excluded from the segmentation. The 3D ventricular heart geometry was then extracted as the image voxels contained between the endocardial and epicardial contours.

### Identification of inFAT and scar on imaging

inFAT was identified as hypoattenuation in the range of −180 Hounsfield units (HU) to −5 HU^42^. Two separate subranges between −180 HU and −50 HU (dense inFAT) and between −50 HU and −5 HU (fat–myocardium admixture) were identified as done in our previous study^42^. All lead and device artifacts were identified by thresholding and were removed from analysis the same as in our previous study^42^. For this study, the term inFAT refers to both dense inFAT and fat–myocardium admixture.

Dense scar and gray zone were identified on LGE-MRI imaging using the full-width half-maximum method as done in our previous studies^41,43^. All voxels with intensity values >50% of the maximal intensity in the myocardium were labeled as dense scar, and voxels with intensity values between 35% and 50% of the maximal intensity were labeled as gray zone. Device, respiratory and motion artifacts were manually identified, annotated and removed from analysis. All regions affected by artifacts on CT or MRI were considered to be non-injured myocardium. In this study, we defined ‘scar’ to be both dense scar and gray zone. All volumes in this study were converted to mass by using a scaling factor of 1.055 g cm^−^^3^ to estimate myocardial tissue density^45^.

### Registration between EAM data and ventricular geometries

To compare the inFAT and scar distributions with clinical procedural data, EAM surface geometries and clinical ablation locations were registered to the patient’s ventricular heart geometry via a rigid transformation, as described in our previous publication^42^. Using open-source Visualization Toolkit system software, EAM surfaces and ablation lesion locations were translated and rotated until they conformed to the heart geometry. The median number of points across EAM surfaces was 13,016 (interquartile range: 11,279–24,922). Points on the EAM surface geometry were projected to the closest point along the heart endocardial or epicardial surface, depending on whether the mapping was performed from the endocardium or epicardium, respectively. All points on the EAM surface geometry that were projected to a point on the heart surface >5 mm away were removed from analysis due to potential for mis-registration. The registration error between EAM surface and ventricular heart geometries was 2.27 ± 1.39 mm (CT geometries: 2.26 ± 1.39 mm, MRI geometries: 2.27 ± 1.39 mm).

### Comparison between EAM data and distribution of inFAT and scar in ventricular geometries

Due to the hemodynamic instability of all patients in this cohort, which is often the case in patients with ischemic heart disease, ablation procedures were performed with substrate-based EAM, meaning that the VT circuit could not be intraprocedurally mapped. Because of this, even though all patients were non-inducible for VT by the end of the ablation procedure, ablation lesions were not necessarily delivered only to sites critical for VT re-entry. Hence, locations of multiple measures indicative of abnormal electrophysiological activity on EAM (low voltage, clinical ablation lesions and DZs) were identified, because these locations represent areas with pro-arrhythmic, abnormal electrophysiological properties that are known to be present at critical VT sites. Voltage abnormalities are the most commonly obtained clinical measurement; they are a hallmark of EAM^1^, and critical VT sites are often found in areas with low voltage. Ablations were selectively delivered to areas with abnormal electrograms that ultimately resulted in non-inducibility of VT, and DZs have been mechanistically linked to critical components of the VT circuitry^20^.

For each EAM point projected to the heart surface of the ventricular geometry, the volume of inFAT and scar was computed within a radius of 1 cm from the EAM point to account for remodeling in the intramural myocardium. Because of the potential for noisy, non-physiological measurements, a threshold was applied for both BiV and UniV to limit the range of values considered for analysis. Specifically, BiV and UniV were capped at 1.5 mV and 8.3 mV, respectively, for analysis, which corresponds to cutoffs used for voltage amplitudes of normal tissue in previous studies^46^. LVZs and MVZs on EAM were defined using both BiV (LVZ ≤ 0.5 mV, 0.5 mV < MVZ < 1.5 mV) and UniV (LVZ ≤ 3.3 mV, 3.3 mV < MVZ < 8.3 mV) cutoffs that were previously reported in the literature^19^. inFAT or scar was defined to be present at an EAM point if it comprised at least 10% of the volume within a 1-cm radius. inFAT_inScar_ was defined as regions with both inFAT and scar. Scar_noFat_ was defined as regions that had scar but no inFAT, whereas inFAT_noScar_ was defined as regions that had inFAT but no scar. Tissue without remodeling was defined as tissue without inFAT and scar.

To assess the overlap of clinical ablation lesions with inFAT and scar, lesion volumes were estimated in all ventricular geometries. Clinical ablations were estimated to burn tissue at a certain radius from the myocardial surface, depending on the type of tissue present at that location. Lesions centered on non-injured myocardium were estimated to encompass a radius of 4.56 mm from the myocardial surface (volume: 397 mm^3^), whereas lesions in gray zone were estimated to encompass a radius of 3.07 mm (volume: 121 mm^3^) and in dense scar a radius of 2.50 mm (volume: 66 mm^3^), corresponding to volumes reported previously in experimental literature^47^. As for dense inFAT and fat–myocardium admixture, there has not been a clear characterization of how radiofrequency ablation penetrates dense inFAT versus fat–myocardium admixture. Limited numerical experiments suggest that thermal lesions successfully created by radiofrequency ablation may be similar between inFAT and scar^48^. Hence, we assumed lesions in dense inFAT and fat–myocardium admixture to be the same as the values reported for scar and gray zone, respectively.

Activation maps acquired during sinus rhythm were also analyzed. Following a previously validated clinical methodology^20^, activation times were binned into eight equally spaced time windows (isochrones) starting from the earliest to the latest activation. The average total activation time across EAMs was 264.4 ± 109.7 ms (average isochrone temporal resolution: 33.0 ± 13.7 ms). Because each isochrone represents a fixed unit of time, more closely spaced isochrones indicate less distance traveled and, hence, a slower conduction speed. To quantify isochronal crowding for each point on the EAM geometry, the number of different isochrones present within a 1-cm radius on the surface EAM geometry was computed. From this, DZs, which are regions of isochronal crowding that have previously been mechanistically linked to critical VT sites^20^, were defined as EAM points with ≥5 isochrones within a 1-cm radius.

Because the arrhythmogenic substrate is inherently 3D and not 2D, DZs reflect electrical propagation through the intramural myocardium, not only along the surface. Thus, to account for the 3D propagation, DZs were estimated to reflect the myocardium within a 1-cm radius of the EAM point. These DZ volumes were then compared with the inFAT and scar distributions.

### Computational ventricular meshes

From the reconstructed ventricular geometries, finite-element, tetrahedral meshes were generated (Materialise Mimics) with ~5 million nodes with an average resolution of ~390 μm to conduct computational simulations using a previously described meshing procedure^49^. This mesh resolution was chosen to maximize computational efficiency and to maintain a sufficient size appropriate for simulating wave propagation, as reported in previous publications^41,42,50^. Furthermore, a previous study indicated that a resolution of ~400 μm is sufficient for avoiding artificial conduction block due to numerical artifacts and accurately identifying the locations of re-entrant activity^49^. Fiber orientations, specific to each individual heart model, were assigned to each computational mesh on a per-element basis, as reported in previous publications^41–43^, using a validated rule-based method^51^.

### Universal ventricular coordinate system for heart models

**Extended Data Fig. 5:**
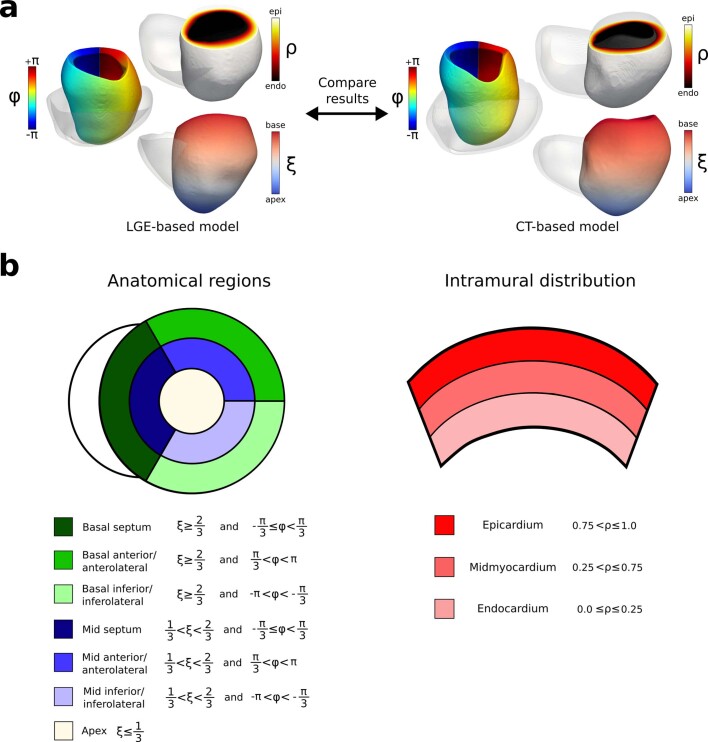
Definitions of the UVC system. **a**: Illustration of the UVC system for both LGE-based and CT-based models. For both CT-based and LGE-based heart ventricular geometries, the UVC system was defined. The three different axes defined were rotational (Ф: blue, cyan, green, yellow, red), transmural (ρ: black, red, yellow, white), and apicobasal (ξ: blue, white, red). **b**: Definitions for anatomical regions and intramural distribution using the UVC system. Seven anatomical regions were defined: basal septum (dark green), basal anterior/anterolateral (green), basal inferior/inferolateral (light green), mid septum (dark blue), mid anterior/anterolateral (blue), mid inferior/inferolateral (light blue) and apex (white). The corresponding UVC ranges are displayed in the legend (left side). The epicardium, midmyocardium, and endocardium were also defined using the UVC system (right). UVC symbols: rotational (Ф), transmural (ρ), and apicobasal (ξ). CT: computed tomography, LGE: late gadolinium-enhanced, UVC: universal ventricular coordinates

For all heart geometries, mesh node coordinates were defined using the universal ventricular coordinate (UVC) system, as described in a previous study^52^. An illustration is shown in Extended Data Fig. 5a. The UVC system describes each myocardial geometry using a common reference space with three axes: apicobasal (apex to base), transmural (endocardium to epicardium) and rotational (counterclockwise starting from the lateral myocardium). With the UVC, myocardial geometries of different shapes and sizes could be compared, and remodeling distributions could be superimposed onto different geometries.

**Extended Data Fig. 6:**
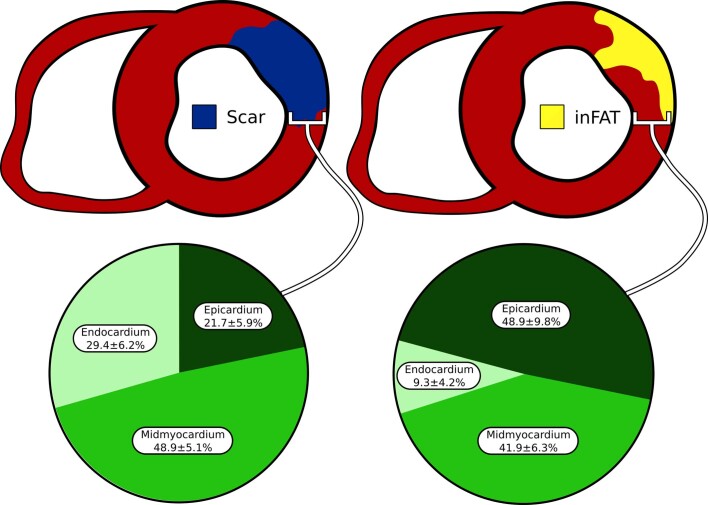
Intramural distribution of inFAT and scar. The percentage of inFAT and scar in the endocardium, mid-myocardium, and epicardium was quantified. inFAT: infiltrating adipose tissue

Using the UVC system, the inFAT and scar distributions were assessed. Definitions for the anatomical regions are presented in Extended Data Fig. 5b. The anatomical regions specified were: basal inferolateral/inferior, basal anterolateral/anterior, basal septal, mid inferolateral/inferior, mid anterolateral/anterior, mid septal and apex (Extended Data Fig. 5b). We also assessed the intramural distribution of inFAT and scar, as shown in Extended Data Fig. 6.

### Incorporation of patient-specific inFAT and scar distributions into heart models

For CT-based heart models, the patient-specific inFAT distribution from CE-CT was incorporated into the ventricular geometry. Similarly, for LGE-based heart models, scar from LGE-MRI was incorporated into the ventricular geometry. Mesh elements in each heart model geometry were each labeled with different tags corresponding to the regions identified on imaging. In the CT-based heart models, there were three region tags: non-injured myocardium, fat–myocardium admixture and dense inFAT. In LGE-based heart models, the three region tags were: non-injured myocardium, gray zone and dense scar.

Hybrid CT-MRI heart models were created by combining the inFAT and scar distributions. The scar distribution from the LGE-based heart models was projected onto the CT-based heart model geometry because CT imaging has a higher resolution and, thus, represents the myocardial structure more accurately. First, the UVCs of mesh elements tagged as dense scar or gray zone on LGE-based heart models were identified. Then, the corresponding UVCs on the CT-based heart models were identified, thus giving each mesh element two distinct tags from each of the CT-based and LGE-based heart models.

**Extended Data Fig. 7:**
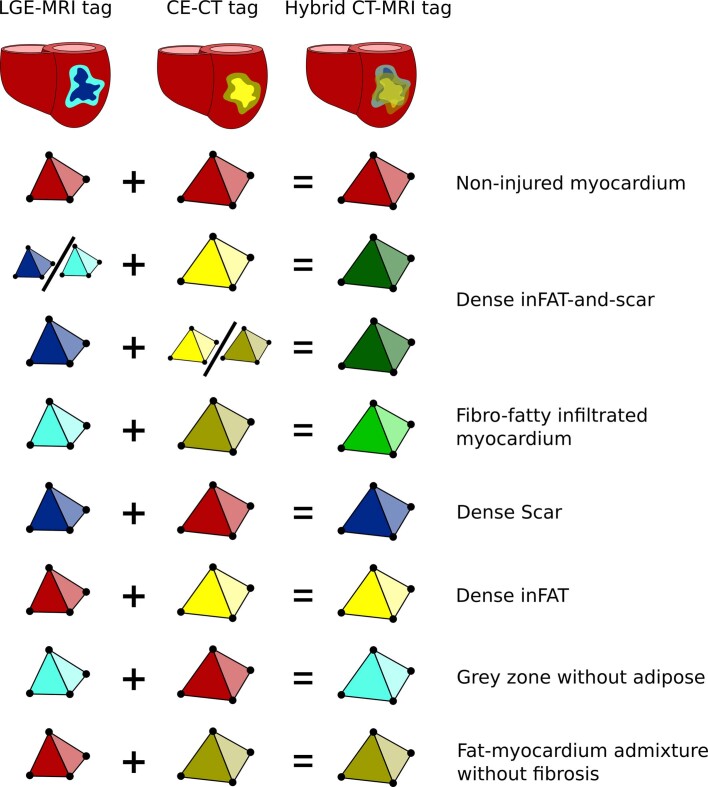
Assignment of mesh elements in hybrid CT-MRI heart models. Seven distinct regions were identified in hybrid CT-MRI heart models based on the corresponding tags in CT- and LGE-based heart models. Colors represents the different possible element tags. CE-CT: contrast-enhanced computed tomography, LGE-MRI: late gadolinium-enhanced magnetic resonance imaging

A summary of how the seven distinct regions were defined in these hybrid CT-MRI models is shown in Extended Data Fig. 7. Non-injured myocardium in hybrid CT-MRI heart models was defined as mesh elements that were tagged as non-injured myocardium in both CT-based and LGE-based heart models. Heterogeneous tissue regions included fat–myocardium admixture without fibrosis, gray zone without adipose and fibrofatty infiltrated myocardium. Mesh elements were tagged as fibrofatty infiltrated myocardium in the hybrid CT-MRI heart models if the tag was fat–myocardium admixture on CT-based heart models and gray zone on LGE-based heart models (Extended Data Fig. 7). Lastly, fully remodeled tissues included dense inFAT only, dense scar only and dense inFAT-and-scar. Any mesh element in hybrid CT-MRI heart models was tagged as dense inFAT-and-scar if there was overlapping adiposity and fibrosis. Mesh elements were tagged as dense inFAT only if there was no fibrosis from the LGE-based heart model, and, conversely, mesh elements were labeled as dense scar only if no inFAT was present (Extended Data Fig. 7).

### Assigning electrophysiology properties in heart models

Ionic properties of the non-injured myocardium were modeled with the ten Tusscher human ventricular myocyte model^53^. Dense inFAT, dense scar and combined dense inFAT-and-scar were modeled as non-conducting insulators, as done in previous works^41–43,50,54–56^. Because there is no proof of fibroblast–myocyte or adipocyte–myocyte coupling in intact hearts, heterocellular coupling was not represented in the models^34,57^. The ionic currents of fat–myocardium admixture, gray zone and fibrofatty infiltrated myocardium regions were adjusted as done in previous works^41–43,50,54–56^. A previous study demonstrated that representing these admixture regions with a heterogeneous structural composition (as opposed to assigning homogeneous electrophysiological properties) did not appreciably impact VT location^58^. Specifically, the ionic currents were scaled according to the experimental literature describing infarct border zone: the peak I_Na_ was reduced by 62%^59^; I_CaL_ was reduced by 69%^60^; I_Kr_ was reduced by 70%; and I_Ks_ was reduced by 80%^61^.

Tissue conductivities for non-injured myocardium in all heart models were assigned a value of 0.08 and 0.00889 S/m in the longitudinal and transverse directions, respectively, the same as used in previous studies^42^. In gray zone regions, transverse conductivity was decreased by 90% to reflect remodeling of gap junctions in infarct border zone^62^. In fat–myocardium admixture, gap junction alterations^15^, decreased conduction velocity^32^ and abnormal electrogram signals^16,28^ are present, similarly to electrophysiological changes in the infarct border zone^63^, but the extent of such changes remains unknown. In the absence of such data, the electrophysiological properties of fat–myocardium admixture were approximated using the same parameters as reported in our previous publication^42^.

Detailed data regarding conduction properties in fibrofatty infiltrated regions in the infarcted myocardium are non-existent. However, regions of concomitant fibrosis and fatty infiltration exhibit greater gap junction alterations than regions of fibrosis or adipose alone^15^, which results in greater conduction slowing. Furthermore, a clinical study found that regions of wall thinning (suggestive of scarring) with inFAT had lower voltage amplitude and prolonged electrogram durations than regions of wall thinning without inFAT, suggesting that these regions exhibit greater electrophysiological abnormalities. Hence, to encapsulate this apparent increased conduction abnormality, we empirically decreased both the longitudinal and transverse conductivities in regions of fibrofatty infiltrated myocardium by an additional 50%, corresponding to an estimated decrease in conduction velocity of 25%.

### Inducing VTs in heart models

A previously validated rapid pacing protocol^43^ was applied from multiple sites to induce all possible VTs in each heart model. Each pacing was delivered to a 1-mm^3^ volume of myocardium. Six stimuli were delivered at 450-ms basic cycle length followed by up to four successive premature stimuli until VT induction. Pacing was delivered from 17 sites, one for each American Heart Association (AHA) segment, as done in a previous work^43^, to comprehensively identify all possible arrhythmias in the patient-specific substrate. Within each AHA segment, pacing sites were projected to be adjacent to the closest inFAT in CT-based heart models and scar in LGE-based heart models to maximize the likelihood of VT induction. For hybrid CT-MRI heart models, the same pacing sites in CT-based heart models were used because the ventricular geometry was the same. In all heart models, if a particular pacing site failed to capture, the stimulus site was manually shifted to nearby conducting tissue. This process was repeated until the stimulus successfully captured. All simulations were performed using Cardiac Arrhythmia Research Package software (https://carp.medunigraz.at/) at a temporal resolution of Δt = 25 μs.

The approach to personalized image-based modeling of VT, as described above, was previously validated with clinical data in multiple studies^41,42,56,64^, demonstrating excellent correspondence between model VT predictions and clinical targets.

### Characterization of VT circuits in heart models

Induced VT was defined as re-entry sustaining at least two cycles at the same critical site, as in previous studies^41–43^. The locations of all VT exit sites were manually selected by visualizing the 3D activation map and choosing the point in a software called meshalyzer (https://github.com/cardiosolv/meshalyzer). VTs were classified as being in the basal, mid or apical myocardium based on the location of the VT exit site (definition in Extended Data Fig. 5b).

**Extended Data Fig. 8:**
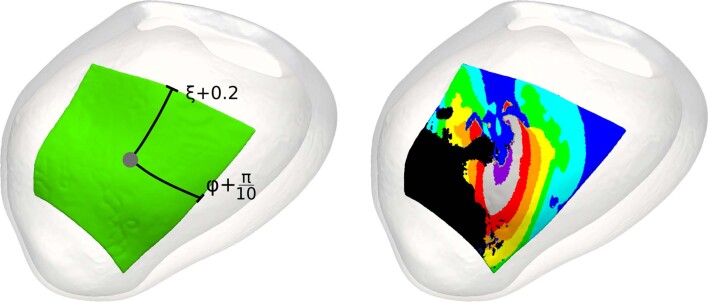
Definition of heart model VT circuits. VT circuits were defined using the UVC system. Letting the grey dot (left panel) denote the UVC corresponding to the manually annotated VT exit site, the VT circuit was defined as all tissues falling within 0.2 units (ξ) in terms of the apicobasal axis, 36° or π/5 radians (Ф) for the rotational axis, and the full transmural myocardium (ρ) for these areas. The activation sequence for a given VT circuit (right panel) from one re-entrant cycle to the next was systematically divided into 8 isochrones: exit site (purple), outer loop (grey and red), entrance (orange), the common pathway (yellow, green, cyan, blue), and the critical isthmus (cyan, blue). UVC: universal ventricular coordinates, VT: ventricular tachycardia.

To define the VT circuit using the UVC system, a window around each VT exit site of 0.4 units for the apicobasal axis, 72° for the rotational axis and the entire myocardium for the transmural axis were used. These values were determined manually and selected to include the entire VT circuit. An illustration of this VT circuit definition is shown in Extended Data Fig. 8. The VT circuit activation sequence was then divided into eight uniformly spaced time windows called isochrones, and these isochrones were used to define the various components of the VT circuit, inspired by the definition used in previous clinical studies^6^. The VT exit is defined to be the first isochrone (purple); the second (gray) and third (red) isochrones reflect the outer loop. The VT entrance is defined as the fourth isochrone (orange), and the fifth (yellow) through eighth (blue) isochrones represent the VT common pathway. The critical isthmus is defined as the sixth (green) and seventh (cyan) isochrones.

**Extended Data Fig. 9:**
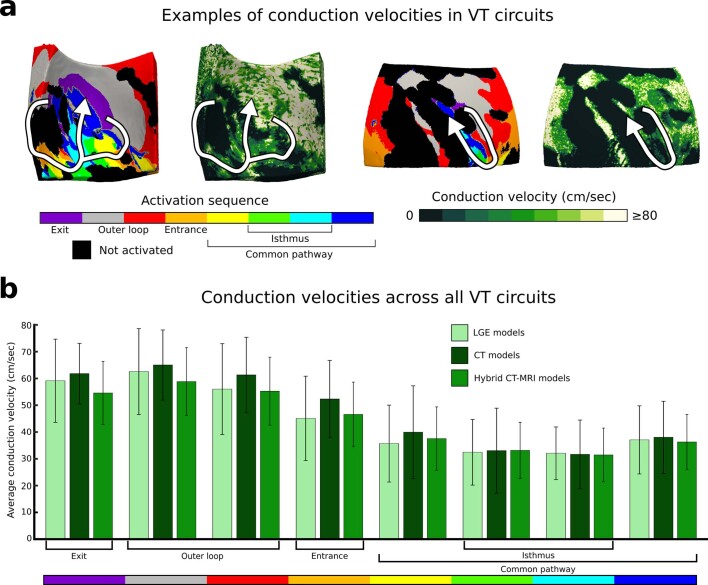
Conduction velocities across model VT circuits. Conduction velocities were measured across VT circuits. **a:** Example of volumetric conduction velocities in two VT circuits in hybrid CT-MRI models. **b:** Conduction velocities in each circuit component in LGE (n = 95 unique VT circuits), CT (n = 97 unique VT circuits), and hybrid CT-MRI models (n = 140 unique VT circuits). Data are presented as mean values ± SD. CT: computed tomography, LGE: late gadolinium-enhanced, MRI: magnetic resonance imaging, VT: ventricular tachycardia.

**Extended Data Fig. 10:**
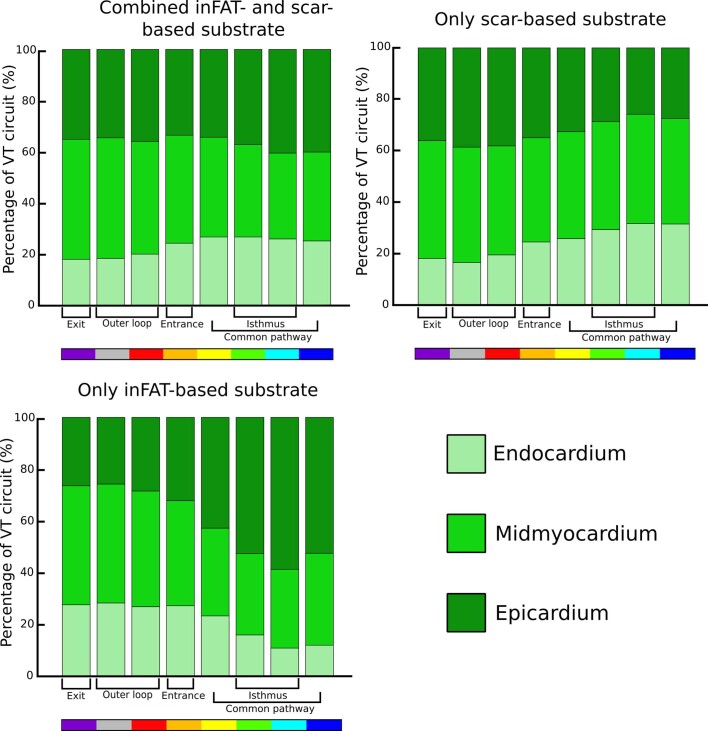
Intramural distribution of VT circuits. The percentage of activation for each VT circuit component was quantified in the endocardium, midmyocardium, and epicardium across all VT circuits. VT: ventricular tachycardia.

At the location of each hybrid CT-MRI model VT circuit, the total amounts of inFAT and scar were measured. The amount of inFAT for VT circuits in LGE-based heart models was measured by superimposing the inFAT distribution from the corresponding CE-CT. To validate whether the conduction properties of computational model VT circuits were representative of that of patient VT circuits, we also measured the total cycle length of each VT (described in Supplementary Results) and conduction velocities within each VT circuit across all models, as shown in Extended Data Fig. 9. These results demonstrate how, despite the altered conduction velocity of fibrofatty infiltrated myocardium, the conduction velocities in the critical VT isthmuses in hybrid CT-MRI models were not significantly different from that of CT-based or LGE-based models (Extended Data Fig. 9). This means that the altered electrical properties of fibrofatty infiltrated regions alone cannot account for differences in arrhythmogenicity between hybrid CT-MRI models and CT-based or LGE-based models. Lastly, we assessed the intramural activation sequence of VT circuits across all models (shown in Extended Data Fig. 10). This figure shows how the inFAT distribution preferentially shifts the activation sequence toward the epicardium.

### Statistical analyses

BiV and UniV of inFAT_inScar_, Scar_noFat_, inFAT_noScar_ and tissue with no remodeling were compared using an unbalanced one-way ANOVA test. The distributions of isochronal crowding between regions of inFAT_inScar_, Scar_noFat_, inFAT_noScar_ and tissue with no remodeling were also compared using an unbalanced one-way ANOVA test. Fisher’s exact tests were used to compare the association between the presence of inFAT_inScar_, Scar_noFat_, inFAT_noScar_ and tissue with no remodeling with the presence of DZs.

To determine the association between the amount of inFAT and scar and the number of VTs induced in the hybrid CT-MRI heart models, a multivariable Poisson regression model was used. To assess differences in arrhythmogenicity between the inFAT-based and scar-based substrates, the total number of VTs in CT-based and LGE-based heart models was assessed with an unpaired *t*-test. To determine if there was a difference in the general distribution of these VTs, unpaired *t*-tests were computed for the number of basal, mid and apical VTs in the CT-based and LGE-based heart models. For VT circuits in LGE-based and hybrid CT-MRI heart models, multivariable linear regression models (adjusted for patient age, infarct age and volume of tissue within the VT circuit) were used to assess whether the amounts of inFAT and/or scar within a VT circuit (in grams) were associated with the volume of clinical ablations and DZs.

### Reporting summary

Further information on research design is available in the Nature Research Reporting Summary linked to this article.

## Supplementary information

Supplementary Information Supplementary Methods and Supplementary Results

Reporting Summary

## Data Availability

Mesh geometries and simulation data are available upon reasonable request to N.A.T. Patient data used in this study cannot be made publicly available without further consent and ethical approval, owing to privacy concerns. The CT and MRI images can be provided by the authors pending Johns Hopkins University IRB and University of Pennsylvania IRB approval and a completed material transfer agreement. Requests for these data should be sent to N.A.T. and/or S.N.

## Code availability

Codes for image processing and simulations are available upon reasonable request to the corresponding author. Custom-written code for analysis is available at https://gitlab.com/natalia-trayanova/fat_infiltration_arrhythmias.
