# Supplementary material for: Fat infiltration in the infarcted heart as a paradigm for ventricular arrhythmias
Source: Nat Cardiovasc Res. 2022 Oct 6;1(10):933–45. doi: 10.1038/s44161-022-00133-6 (PMC9802586; doi:10.1038/s44161-022-00133-6)
Supplement: Supplementary file 2 — Reporting Summary [file 44161_2022_133_MOESM2_ESM.pdf]

nature portfolio

Corresponding author(s): Natalia A. Trayanova

Last updated by author(s): Aug 5, 2022

Reporting Summary

Nature Portfolio wishes to improve the reproducibility of the work that we publish. This form provides structure for consistency and transparency in reporting. For further information on Nature Portfolio policies, see our Editorial Policies and the Editorial Policy Checklist.

Statistics

For all statistical analyses, confirm that the following items are present in the figure legend, table legend, main text, or Methods section.

n/a	Confirmed	
[ ]	[x]	The exact sample size (*n*) for each experimental group/condition, given as a discrete number and unit of measurement
[x]	[ ]	A statement on whether measurements were taken from distinct samples or whether the same sample was measured repeatedly
[ ]	[x]	The statistical test(s) used AND whether they are one- or two-sided<br>*Only common tests should be described solely by name; describe more complex techniques in the Methods section.*
[ ]	[x]	A description of all covariates tested
[x]	[ ]	A description of any assumptions or corrections, such as tests of normality and adjustment for multiple comparisons
[ ]	[x]	A full description of the statistical parameters including central tendency (e.g. means) or other basic estimates (e.g. regression coefficient) AND variation (e.g. standard deviation) or associated estimates of uncertainty (e.g. confidence intervals)
[ ]	[x]	For null hypothesis testing, the test statistic (e.g. *F*, *t*, *r*) with confidence intervals, effect sizes, degrees of freedom and *P* value noted<br>*Give P values as exact values whenever suitable.*
[x]	[ ]	For Bayesian analysis, information on the choice of priors and Markov chain Monte Carlo settings
[x]	[ ]	For hierarchical and complex designs, identification of the appropriate level for tests and full reporting of outcomes
[x]	[ ]	Estimates of effect sizes (e.g. Cohen's *d*, Pearson's *r*), indicating how they were calculated

Our web collection on statistics for biologists contains articles on many of the points above.

Software and code

Policy information about availability of computer code

Data collection	Electroanatomic mapping data was acquired from the commercial Biosense Webster CARTO 3 system Version 6 available at our institution. Imaging data was acquired through our institution's PACS server as raw DICOMs. Because these represent primary clinical data, appropriate permissions will have to be obtained in accordance with our institutional review board for data access.
Data analysis	3D Slicer version 4.8 (open-source visualization toolkit software) was used for image processing and resampling images into short axis. CardioViz3D version 1.5 (https://download.cnet.com/CardioViz3D/3000-2054_4-10834669.html) was used for segmenting the myocardium (details in Methods section). Custom written code in MATLAB Version 2021A was used to generate the segmentation masks by using the outputs from the CardioViz3D software. Finite element heart mesh geometries were created using the Materialise Mimics software. Whole-heart electrophysiological simulations were conducted using the Cardiac Arrhythmia Research Package (CARP) software (custom-written solver in C code for solving finite element systems). Results could be reproduced using the equivalent, publicly available, newer openCARP software (https://opencarp.org). Simulations were visualized using a program called meshalyzer (https://github.com/cardiosolv/meshalyzer). Figures of mesh geometries were generated using Paraview V5.6 (https://www.paraview.org/). All codes for segmentation, mesh generation, and simulations were described and presented in previous publications. All analyses for the current study were performed using custom-written code in MATLAB Version 2021A. The analysis MATLAB codes are available at https://gitlab.com/natalia-trayanova/fat_infiltration_arrhythmias

For manuscripts utilizing custom algorithms or software that are central to the research but not yet described in published literature, software must be made available to editors and reviewers. We strongly encourage code deposition in a community repository (e.g. GitHub). See the Nature Portfolio guidelines for submitting code & software for further information.

Data

Policy information about availability of data

All manuscripts must include a data availability statement. This statement should provide the following information, where applicable:

- Accession codes, unique identifiers, or web links for publicly available datasets
- A description of any restrictions on data availability
- For clinical datasets or third party data, please ensure that the statement adheres to our policy

Mesh geometries and simulation data are available upon reasonable request to N.A.T. Patient data used in this manuscript cannot be made publicly available without further consent and ethical approval, owing to privacy concerns. The CT and MRI images can be provided by the authors pending Johns Hopkins University institutional review board and University of Pennsylvania institutional review board approval and a completed material transfer agreement. Requests for these data should be sent to N.A.T. and/or S.N.

Human research participants

Policy information about studies involving human research participants and Sex and Gender in Research.

Reporting on sex and gender	Gender-based analysis was not relevant to the current study. Our modeling approach also does not account for sex and gender differences in post-infarct ventricular electrophysiology as there is no currently available data that describes these differences well. Furthermore, a gender-based analysis is unlikely to yield significant results because patients with ischemic cardiomyopathy who suffer from VT are overwhelmingly male (Sapp et. al 2016 doi: 10.1056/NEJMoa1513614).
Population characteristics	All patients had a history of myocardial infarction and ventricular tachycardia. The mean age was 71.9 +/- 10.6 years (shown in the supplementary materials).
Recruitment	Data was obtained from patients enrolled as part of the ongoing prospective INFINITY study. In this study, patients were undergoing a ventricular tachycardia (VT) ablation and had a history of myocardial infarction. Their enrollment meant the dual acquisition of cardiac MRI and cardiac CT. All patients were appropriately informed about the purpose of the study and the risks involved prior to consenting to being enrolled. Although our study was performed from two-centers, there may be an unintentional sampling bias as both institutions are within similar geographic locations of the United States which may limit generalizability of our results to international patient cohorts.
Ethics oversight	All data was acquired under the guidance of our institutional review boards (Study #831270) of Johns Hopkins University and University of Pennsylvania.

Note that full information on the approval of the study protocol must also be provided in the manuscript.

Field-specific reporting

Please select the one below that is the best fit for your research. If you are not sure, read the appropriate sections before making your selection.

☒ Life sciences ☐ Behavioural & social sciences ☐ Ecological, evolutionary & environmental sciences

For a reference copy of the document with all sections, see nature.com/documents/nr-reporting-summary-flat.pdf

Life sciences study design

All studies must disclose on these points even when the disclosure is negative.

Sample size	24 patients (13 from University of Pennsylvania and 11 from Johns Hopkins) were recruited sequentially from the span of 2019 to 2021. This sample size was chosen based on availability during the recruitment interval. Although no sample size calculation was used, this sample size was deemed sufficient because this study, to date, has the largest cohort of post-infarct VT patients with both CE-CTs and LGE-MRIs. Furthermore, most patient-specific, computational modeling studies performing the same detailed-level of analysis as performed in this study involve significantly less patients (n<10).
Data exclusions	Patients with imaging data of insufficient quality were excluded from this study. Reasons for insufficient quality included: poor image resolution, poor delineation of blood pool from myocardium, breathing artifacts, motion artifacts, and missing data. From this criteria, only 24 patients were deemed to have sufficiently high-quality CTs and MRIs in this enrollment period and were thus included in the study.
Replication	The results of our computational models are deterministic and all the codes, and data files used to reproduce the simulations are available upon reasonable request. The analysis codes are available at https://gitlab.com/natalia-trayanova/fat_infiltration_arrhythmias.
Randomization	Randomization was not applicable because patients were recruited sequentially as part of this prospective cohort study. All patients received the same VT ablation treatment, and both CTs and MRIs were acquired as part of the study requirements.
Blinding	Simulations were performed blinded to the electroanatomical mapping data. Only once simulations were completely finished was the

mapping and simulation data analyzed.

Reporting for specific materials, systems and methods

We require information from authors about some types of materials, experimental systems and methods used in many studies. Here, indicate whether each material, system or method listed is relevant to your study. If you are not sure if a list item applies to your research, read the appropriate section before selecting a response.

Materials & experimental systems

n/a	Involved in the study
☒	☐ Antibodies
☒	☐ Eukaryotic cell lines
☒	☐ Palaeontology and archaeology
☒	☐ Animals and other organisms
☒	☐ Clinical data
☒	☐ Dual use research of concern

Methods

n/a	Involved in the study
☒	☐ ChIP-seq
☒	☐ Flow cytometry
☒	☐ MRI-based neuroimaging
