# Supplementary material for: Fat infiltration in the infarcted heart as a paradigm for ventricular arrhythmias
Source: Nat Cardiovasc Res. Author manuscript; Available in PMC 2022 Dec 30. (PMC9802586; doi:10.1038/s44161-022-00133-6)
Supplement: Supplementary file 1 [file NIHMS1843489-supplement-supplementary_materials.docx]

**Supplementary Information**

**Title: Fat Infiltration in Infarcted Hearts as an Unexplored Paradigm for Ventricular Arrhythmias**

**Authors:** Eric Sung BA^1,3^, Adityo Prakosa PhD^1,3^, Shijie Zhou PhD^1,3^, Ronald D. Berger MD/PhD^2,3^, Jonathan Chrispin MD^2,3^*, Saman Nazarian MD/PhD^4^*, Natalia A. Trayanova PhD^1,3^*

1 Department of Biomedical Engineering, Johns Hopkins University, Baltimore, MD, USA

2 Section of Cardiac Electrophysiology, Division of Cardiology, Department of Medicine, Johns Hopkins Hospital, Baltimore, MD, USA

3 Alliance for Cardiovascular Diagnostic and Treatment Innovation, Johns Hopkins University, Baltimore, MD, USA

4 Division of Cardiology, Perelman School of Medicine, University of Pennsylvania, Philadelphia, PA, USA

****Equal contribution senior authors*** 

# Supplementary Methods

**Baseline Patient Characteristics**

	The mean patient age was 71.9±10.6 years. The mean infarct age was 18.8±14.5 years. All patients were on beta blockers and at least one anti-arrhythmic medication (amiodarone or sotalol). 

**Ablation Procedure Characteristics**

	Substrate-based ablation was performed for all patients enrolled in this study. Under sedation, non-invasive programmed stimulation was performed through the patient's implanted cardioverter defibrillator to identify the primary VT morphology. Under general anesthesia or moderate sedation, access to the left ventricle was achieved via a retrograde aortic or trans-septal approach. If epicardial mapping was deemed necessary, pericardial access was obtained via a percutaneous subxiphoid puncture. Electroanatomical mapping in sinus rhythm was performed with a multipolar catheter (Penta Ray or Deca Nav) or irrigated tip ablation catheter. Ablation was performed using 35-50 watts in specific areas consistent with the critical isthmus along with areas of abnormal electrophysiological activity (fractionated potentials, late potentials, late abnormal ventricular activities, etc). The procedure endpoint was elimination of all VTs induced with programmed stimulation at two drive cycle lengths, up to triple extra-stimuli.

**Analysis of Intramural myocardium**

The intramural inFAT and scar was assessed in the endocardium, midmyocardium, and epicardium using the UVC definitions (Extended Data Fig.7). The percentage of activation for each VT circuit component distributed in the endocardium, midmyocardium, and epicardium was also computed.

**Measurement of Intramural Conduction Velocity**

Volumetric conduction velocity (CV) was measured intramurally using the triangulation technique extrapolated for 3D, presented in detail in a prior publication.^1,2^ For each triangular face in a tetrahedral element, the triangulation technique was applied to estimate the conduction speed and direction. Once the CVs were computed for each triangular face, the tetrahedral CV was computed as the sum of CVs across the 4 triangular faces. Ad hoc conditions were employed to remove any non-physiological CVs. Specifically adjacent activation times of >10 ms, which likely indicated locations of conduction block, and <0.25 ms, which likely indicate numerical errors, were removed from analysis to obtain a more physiological range of CVs. 

# Supplementary Results

**Intramural distribution of inFAT versus scar**

Extended Data Figure 6 shows the intramural distribution of the scar and inFAT. Scar localized mostly to the midmyocardium (48.9±5.1%) and the endocardium (29.4±6.2%), but slightly less to the epicardium (21.7±5.9%). In contrast, inFAT was located primarily in the epicardium (48.9±9.8%) and the midmyocardium (41.9±6.3%), but very little localized to the endocardium (9.3±4.2%). These results demonstrate that the inFAT and scar distributions exhibit differences in the intramural myocardium and that inFAT may have a role in epicardial post-infarct substrate.

**Conduction properties of VT circuits across models**

	We assessed the electrical conduction characteristics in the LGE-based, CT-based, and hybrid CT-MRI models. The average tachycardia cycle length (TCL) was 309.5±36.1 ms for VTs induced in the hybrid CT-MRI models. VTs induced in the CT-based models did not have significantly different TCLs than VTs in the LGE-based models alone (inFAT vs scar: 310.0±36.8 ms vs. 312.4±33.8 ms, p=0.63). The TCLs of these model VT circuits are consistent with the TCLs of clinical VT circuits previously reported in the literature.^3^

Extended Data Figure 9a provides examples of VT circuits and corresponding conduction velocity (CV) measurements in the hybrid CT-MRI models. In all models, conduction velocity was highly heterogeneous throughout the VT circuit. Extended Data Figure 9b shows a comparison of the CVs measured throughout the VT circuit across the different models. In the hybrid CT-MRI models, CVs tended to be slower in the common pathway (34.6±10.9 cm/s) and critical isthmus (32.3±10.3 cm/s) than in the exit site (54.5±11.8 cm/s), the outer loop (57.0±12.8 cm/s), and the entrance (46.5±12.0 cm/s), consistent with prior clinical studies.^3–5^ Components of the VT circuits in the CT- and LGE-based models had similar CVs for the exit (61.7±11.3 vs 59.1±15.5 cm/s), outer loop (63.1±13.2 vs 59.3±15.1 cm/s), the common pathway (35.6±10.8 vs 34.3±9.1 cm/s) and the critical isthmus (32.3±12.6 vs 32.3±9.6 cm/s), except for the entrance (52.3±14.4 vs 45.1±15.7 cm/s, p<0.05). Collectively, our results demonstrate that the conduction properties of VT circuits in our computational models are consistent with those measured in clinical studies.

**The critical isthmus of VT circuits in the inFAT-based substrate tend to localize more towards the epicardium than those in the scar-based substrate.**

Because of the importance of intramural substrate, we also characterized the 3D structure of the VT circuits. Extended Data Figure 10 shows the intramural distribution of VT circuit activations in the combined inFAT-and-scar substrate (left), only scar-based substrate (middle), and only inFAT-based substrate (right). The critical isthmuses of VT circuits in the combined inFAT-and-scar were not evenly distributed; 38.8±27.0% of the activation sequence localized to the epicardium, 26.3±23.9% to the endocardium, and 34.9±19.7% to the midmyocardium (Extended Data Fig.10, left). When assessing the individual scar-based substrate, the critical isthmus was distributed intramurally with 27.3±19.5% of activations in the endocardium, 30.5±19.7% in the epicardium, and 42.2±14.6% in the midmyocardium (Extended Data Fig.10, middle). However, the critical isthmuses in the inFAT-based substrate had a greater proportion of activation in the epicardium (55.7±31.9%) versus the midmyocardium (30.9±22.2%) and the endocardium (13.4±16.8%) (Extended Data Fig.10, right). These results means that inFAT, which localizes more to the epicardium and midmyocardium, likely contributes to epicardial substrate arrhythmogenicity. 

# References

1.	Good, W. W. *et al.* Estimation and Validation of Cardiac Conduction Velocity and Wavefront Reconstruction Using Epicardial and Volumetric Data. *IEEE Transactions on Biomedical Engineering* 1–1 (2021) doi:10.1109/TBME.2021.3069792.

2.	Good, W. W. *et al.* Quantifying the spatiotemporal influence of acute myocardial ischemia on volumetric conduction velocity. *Journal of Electrocardiology* **66**, 86–94 (2021).

3.	Nishimura, T. *et al.* Circuit Determinants of Ventricular Tachycardia Cycle Length. *Circulation* 212–226 (2021) doi:10.1161/CIRCULATIONAHA.120.050363.

4.	Aronis, K. N. *et al.* Accurate Conduction Velocity Maps and Their Association With Scar Distribution on Magnetic Resonance Imaging in Patients With Postinfarction Ventricular Tachycardias. *Circulation: Arrhythmia and Electrophysiology* **13**, 326–335 (2020).

5.	Frontera, A. *et al.* Outer loop and isthmus in ventricular tachycardia circuits: Characteristics and implications. *Heart Rhythm* **17**, 1719–1728 (2020).
